# Sensory manipulation results in increased dorsolateral prefrontal cortex activation during static postural balance in sedentary older adults: An fNIRS study

**DOI:** 10.1002/brb3.1109

**Published:** 2018-09-19

**Authors:** Wei‐Peng Teo, Alicia M. Goodwill, Ashlee M. Hendy, Makii Muthalib, Helen Macpherson

**Affiliations:** ^1^ Institute for Physical Activity and Nutrition (IPAN) Deakin University Burwood Vic. Australia; ^2^ School of Psychology Australian Catholic University Melbourne Vic. Australia; ^3^ Silverline Research Brisbane QLD Australia

**Keywords:** aging, neuroimaging, postural control, prefrontal cortex, sensory orientation test

## Abstract

**Background:**

The dorsolateral prefrontal cortex (DLPFC) is involved with allocating attentional resources to maintain postural control. However, it is unknown whether age‐related structural and functional declines of the DLPFC may impair postural control during sensory manipulation. In this study, we aim to understand the effects of aging on the DLPFC when sensory cues were removed or presented inaccurately (i.e., increased sensory complexity) during the sensory orientation test (SOT).

**Methods:**

Twenty young (18–25 years) and 18 older (66–73 years) healthy adults were recruited to undertake the SOT, which consisted of six conditions aimed at removing or disrupting the visual, vestibular, and proprioceptive senses. During these six SOT conditions, functional near‐infrared spectroscopy (fNIRS), consisting of eight transmitter‐receiver optode pairs (four channels over the left and right DLPFC), was used to measure hemodynamic responses (i.e., changes in oxy‐ [O_2_Hb] and deoxyhemoglobin [HHb]) from the bilateral DLPFC.

**Results:**

Our results show an increase in bilateral DLPFC activation (i.e., increase in O_2_Hb and concomitant smaller decrease in HHb) with increasing sensory complexity in both young and older adults. The increase in left and right DLPFC activation during more complex sensory conditions was greater, which was concomitant with reduced balance performance in older adults compared to younger adults. Furthermore, we observed a right lateralized DLPFC activation in younger adults. Finally, a significant positive association was observed between balance performance and increased bilateral DLPFC activation particularly for SOT conditions with greater sensory disruptions.

**Conclusion:**

Our findings highlight the involvement of the DLPFC in maintaining postural control, particularly during complex sensory tasks, and provide direct evidence for the role of the DLPFC during postural control of a clinically relevant measure of balance.

## 1 INTRODUCTION

Human postural control is reliant on visual, vestibular, and proprioceptive senses to maintain a stable upright stance (Blumle, Maurer, Schweigart, & Mergner, 2006). In particular, aging is accompanied by a reduced capacity to respond to external perturbations during postural control, particularly when sensory cues are hindered or inaccurate (de Dieuleveult, Siemonsma, van Erp, & Brouwer, 2017). This is in part due to an age‐related decline in neuromuscular function (i.e., changes in muscle morphology and neuromuscular transmission; Clark et al., 2010; Gomes, Reis, Neves, Petrella, & de Abreu, 2013; Takacs, Carpenter, Garland, & Hunt, 2013) and reduced cognitive and visuospatial processing abilities (Horak, 2006; Mihara, Miyai, Hatakenaka, Kubota, & Sakoda, 2008). Accordingly, biomechanical analyses of static posturographic measures, such as those obtained from the sensory organization test (SOT) have provided strong evidence for a shift in sensory utilization during postural control with aging (Camicioli, Panzer, & Kaye, 1997; Cohen, Heaton, Congdon, & Jenkins, 1996).

While understanding the biomechanical changes during sensory manipulation provide some insight on how postural control alters with age, little is known about how the brain is involved in processing sensory cues to maintain upright posture. Utilizing a top‐down approach, Allali et al. (2014) used functional magnetic resonance imaging (fMRI) to show age‐related differences in cortical activation patterns in the right orbitofrontal cortex and left dorsolateral prefrontal cortex (DLPFC) during an imagined locomotion task. However, the ecological validity of using an imagined task during fMRI is questionable as it only provides an implied measure of brain response during actual upright body movements or postural control. Recent systematic reviews have highlighted the ability to apply fNIRS as a neuroimaging technique to quantify the cortical control of static and dynamic forms of balance and gait (Herold, Wiegel, et al., 2017; Wittenberg, Thompson, Nam, & Franz, 2017). In this context, fNIRS may offer an advantageous alternative to fMRI due to reduced physical constraints and greater portability (Herold, Wiegel et al., 2017; Wittenberg et al., 2017). The concept of fNIRS as well as fMRI is based on the assumption that brain activity leads to an increase in regional cerebral blood flow due to neurovascular coupling mechanisms, which is reflected in the fNIRS hemodynamic response signal by an increase in oxyhemoglobin [O_2_Hb] and decrease in deoxyhemoglobin [HHb] concentration levels (Ferrari & Quaresima, 2012). Karim, Fuhrman, Sparto, Furman, and Huppert (2013) were the first to successfully use fNIRS to measure cortical hemodynamic responses from the frontal, temporal, and parietal regions of the brain in young healthy participants during the SOT. Their results showed bilateral activation in the temporal–parietal areas (i.e., superior temporal and supramarginal gyri) when both vision and proprioceptive information were degraded. Greater cortical activation in the primary sensorimotor cortex (pre‐ and postcentral gyri) and supplementary motor area during an unstable standing task (i.e., balance board) has also since been demonstrated in young healthy adults (Herold, Orlowski, Bormel, & Muller, 2017). Employing a similar fNIRS paradigm with the SOT as Karim et al. (2013), Lin, Barker, Sparto, Furman, and Huppert (2017) showed an increase in frontal‐parietal and occipital activation when vestibular and visual information were used predominantly during the SOT in older adults compared to middle‐aged adults. However, no differences in postural sway between middle‐aged and older adults were shown. Taken together, these studies provide strong evidence for an age‐related difference in frontal–parietal cortical activation during postural control that can be measured ecologically with fNIRS.

From a motor control perspective, the DLPFC has been implicated in postural control and balance (Fujita, Kasubuchi, Wakata, Hiyamizu, & Morioka, 2016; Mihara et al., 2008), such that the DLPFC is involved with integrating sensory cues and allocating attentional resources during postural control (Woollacott & Shumway‐Cook, 2002). Particularly related to aging, recent fNIRS studies have shown an age‐related reduction in effective (Huo et al., 2018) and functional connectivity (Wang et al., 2016) between the left and right DLPFC during postural changes between older and young adults. However, these studies did not employ clinical measures such as the SOT to manipulate the presentation of sensory cues. While preliminary data have shown differences in frontal cortical activation during the SOT between middle‐aged and older adults (Lin et al., 2017), age‐related differences in DLPFC activation between young and older adults relevant to the SOT have not been examined.

In this context, we investigated the age‐related differences in bilateral DLPFC activation during sensory manipulation of a balance task (SOT) in healthy young and older adults. Unlike the study by Lin et al. (2017) that used a frontal–parietal optode montage, we employed an eight‐channel optode montage to optimize coverage specific to the left and right DLPFC (_L_DLPFC and _R_DLPFC). We hypothesized that older adults would experience greater bilateral DLPFC activation during the SOT conditions where sensory cues are removed or perturbed. These findings will provide direct evidence for the role of the DLPFC during postural control of a clinically relevant measure of balance and enhance our understanding of the cortical mechanisms associated with balance in older adults.

## 2 METHODS

### 2.1 Participants

Twenty young (aged 18‐25 years, 10M/10F) and 18 older (aged 66‐73 years, 10M/8F) sedentary, otherwise healthy, adults were recruited. Participants were excluded if they (a) scored less than 25 on the Mini‐Mental State Examination (MMSE); (b) had a known vestibular impairment; (c) had a visual impairment not rectified by wearing a visual aid (e.g., glasses); (d) suffered from a lower‐limb musculoskeletal injury in the last 6 months; (e) had a previous diagnosis of acquired brain injury or stroke; (f) were on medication for any mood or neurological disorders; (g) suffer severe motion sickness; (h) prone to fainting; and (i) had a known diagnosis of any neurodevelopmental or neurodegenerative disorder. Written informed consent was obtained from all participants. All procedures were performed in accordance with the ethical standards of the Deakin University human research ethics committee (2016‐045) and the Declaration of Helsinki.

### 2.2 SOT setup and data processing

The Neurocom Balancemaster system (Neurocom International Inc, Oregon, USA) was used to SOT, which comprised of six different sensory conditions in quiet stance, and measured the center of pressure (COP) path in centimeters (cm). The test began with condition 1 (eyes open in a static stance), and sequentially moves through each condition which progressively becomes more challenging by either distracting or removing visual and/or proprioceptive feedback. The visual surround and force platform were sway‐referenced, which referred to the tilting of the support surface and/or visual surround in response to movement of the participant's COP. If a participant has poor anterior–posterior control over their COP, the force plate and visual surround will move correspondingly in an anterior–posterior orientation. During the sway‐referenced conditions, erroneous sensory information was presented in an attempt to disconcert the participant, while the force platform measured the participant's ability to compensate by using other senses to maintain balance equilibrium. Three 20‐s trials were conducted for each sensory condition with an intertrial rest period of 60 s. During each rest period, all participants were allowed to readjust their foot position if necessary and was asked to remain standing still upright for at least 30 s with their eyes opened and focused onto a fixation cross at eye‐level in front of them. The sensory conditions included the following:

- Condition 1 (COND1)—Eyes open, unmoving surface, and visual surround;
- Condition 2 (COND2)—Eyes closed, unmoving surface;
- Condition 3 (COND3)—Eyes open, unmoving surface, and sway‐referenced visual surround;
- Condition 4 (COND4)—Eyes open, sway‐referenced surface, and fixed visual surround;
- Condition 5 (COND5)—Eyes closed, sway‐referenced surface;
- Condition 6 (COND6)—Eyes open, sway‐referenced surface, and visual surround.

Following each trial, an equilibrium score was computed, which quantified postural stability during each trial of the six sensory conditions. The equilibrium score compared the participant's anterior–posterior sway during each trial to a theoretical sway stability limit of 12.5 degrees. A participant swaying to the limits of stability will receive a low score, while a score closer to 100 indicates good stability and minimum sway. To quantify postural control for each sensory condition, the averaged equilibrium score across three trials within each sensory condition was used.

### 2.3 FNIRS setup, data collection, and processing

**Figure 1:**
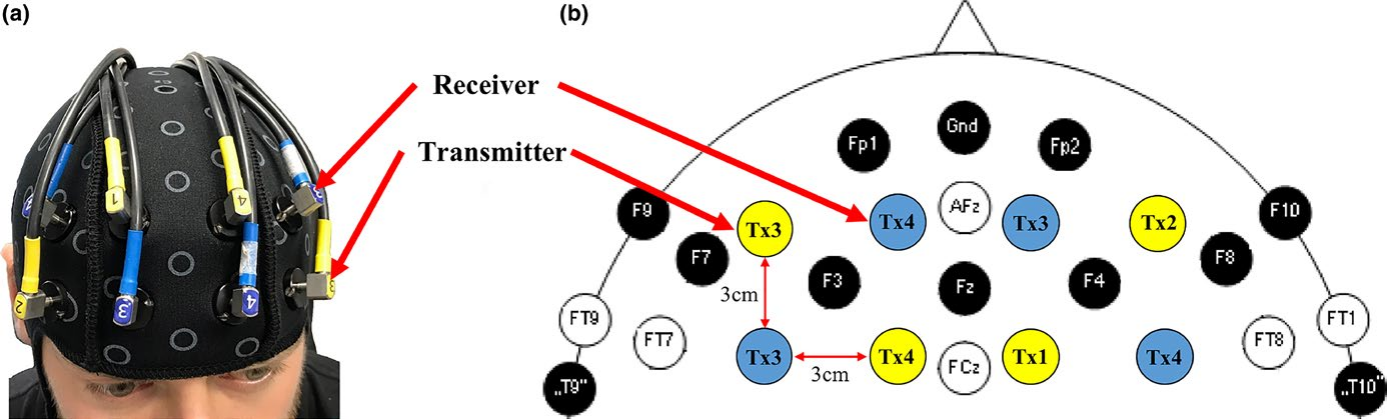
An illustration of the (a) location and setup of eight channels Tx–Rx optode montage on the left and right DLPFC and (b) location of the Tx and Rx in relation to a 10–20 electroencephalography electrode setup

The fundamental basis for fNIRS is that most biological tissue such as skin and bone are transparent to NIR light, while O_2_Hb and HHb are better absorbers of NIR light in the spectrum of 700–900 nm (Isac & Matthes, 1938; Isac, Matthes, & Yamanaka, 1938; Matthes, 1938; Severinghaus, 2007). The principle of fNIRS is based on the absorption rate of O_2_Hb and HHb using the modified Beer–Lambert law (MBLL) (Delpy et al., 1988) at two different wavelength of NIR light between transmitter (Tx) and receiver (Rx) pairs placed over the scalp. Therefore, brain maps can be developed by spatially arranging the NIR light Tx–Rx pairs on the head so that specific brain region(s) of interest can be determined (Boas, Dale, & Franceschini, 2004). In this study, an eight‐channel continuous‐wave NIRS system (Oxymon Mk III, Artinis Medical Systems, The Netherlands) was used to measure fNIRS signals using two wavelengths at 750 and 850 nm during the six sensory conditions of the SOT. Hemodynamic responses were measured from the bilateral DLPFC using four pairs of Tx and Rx over the forehead region (Figure 1). Each receiver measures time‐multiplexed NIR light from two transmitter sources located at a distance of 3 cm, resulting in a total of eight channels (four channels on the left and right DLPFC) of O_2_Hb and HHb. For each participant, an individual differential path‐length factor (DPF) was used to account for age differences according to Scholkmann and Wolf (2013). All signals were sampled at a rate of 10 Hz. Figure 1 shows the placement of the Tx and Rx over the forehead. To identify the functional brain region of interest in which the fNIRS optodes are measuring from, we employed a MATLAB‐based toolbox (fOLD—fNIRS optode location decider) as reported by Zimeo Morais, Balardin, and Sato (2018). The fOLD toolbox provides an accurate estimate of spatial brain mapping of electroencephalography (EEG) 10–20 systems on associated Brodmann areas (Rorden & Brett, 2000). Based on this method, we have identified the _L_DLPFC and _R_DLPFC as the regions of interest in this study.

Measures of O_2_Hb and HHb from all eight channels were exported to and processed using HOMER2, a MATLAB‐based toolbox (MATLAB 2015b, The MathWorks Inc, Massachusetts, USA) to analyze fNIRS data (Huppert, Diamond, Franceschini, & Boas, 2009). As described by Brigadoi et al. (2014), all raw data were first converted to changes in optical density (OD). Channels with low signal‐to‐noise ratio were removed using the enPruneChannels function. As all channels displayed excellent signal‐to‐noise ratio, none of the channels were excluded from the analysis. A motion artifact detection algorithm (hmrMotionArtifactByChannel; AMPThresh = 0.40, SDThresh = 5.0, tMotion = 0.5, and tMask = 1.0) was then used to identify motion artifacts on a channel‐by‐channel basis that exceed the prespecified thresholds in the OD time series. After motion artifact identification was performed, a motion artifact correction method using the principal component analysis (PCA) filter was applied to any active channel that exhibited a signal change greater than the prespecified thresholds (hmrMotionArtifactPCArecurse; AMPThresh = 0.40, SDThresh = 5.0, tMotion = 0.5, tMask = 1.0, nSV = 0.97, and maxlter = 5). A bandpass filter (hmrBandpassFilt; high‐pass = 0.01 and low‐pass = 0.50) was then applied to filter out low‐ or high‐frequency noise. The OD data were then converted into concentration changes using the MBLL (Delpy et al., 1988) using the hmrOD2Conc function (ppf = 6.0, 6.0) and block averaged (hmrBlockAvg; trange = −5.0, 25.0) to provide an indication of average change of three trials for all eight channels during each sensory condition. A figure of the data processing pipeline may be found in the Supporting Information Appendix S1 (Supporting Information Figure S1). The change in hemodynamic response for the _L_DLPFC (four channels) and _R_DLPFC (four channels) was averaged to determine hemispheric differences in cortical activation between young and older adults. Additionally to determine hemispheric lateralization, a laterality index of O_2_Hb and HHb, as described by Seghier (2008) for fMRI studies, was calculated for all conditions in young and older adults. The formula used to calculate the laterality index is as follows:

$$\text{Laterality}\;\text{index}=\frac{\text{Left}\;\text{DLPFC}\;-\;\text{Right}\;\text{DLPFC}}{\text{Left}\;\text{DLPFC}+\text{Right}\;\text{DLPFC}}$$

Based on the formula above, negative values up to −1 indicated _R_DLPFC dominance, while positive values up to +1 indicated _L_DLPFC dominance. An overall average of all eight channels was also calculated to provide an overall measure of DLPFC activation during each sensory condition. While O_2_Hb measures are typically used to represent cerebral activation, it is also sensitive to systemic changes in blood pressure, skin blood flow, and heart rate compared to HHb (Kirilina et al., 2012). For this reason, we considered changes in both O_2_Hb and HHb as true indicators of cerebral activation.

### 2.4 Experimental design

Participants attended one testing session lasting approximately 90 minutes at the Clinical Exercise Learning Centre, Deakin University. Prior to the testing session, individual demographical characteristics such as height (kg), weight (cm), education status (years of formal education), and the MMSE was taken. An fNIRS headcap was fitted, and the participants were harnessed onto the Balancemaster system. The harness was tightened to a point that prevented falling but did not restrict anterior–posterior movement. To prevent any practice effect that may influence balance and hemodynamic responses, only one practice trial for each sensory condition was performed. Upon completion of the practice trials, the testing session commenced with all sensory conditions sequentially performed. To exclude the potential for an order effect on hemodynamic response, a subsample of five young participants repeated the test on a separate day with the order of sensory condition presented randomly.

### 2.5 Statistical analysis

Data were screened for normality using histograms and Shapiro–Wilks test. Independent *t* tests were used to compare demographics between young and older adults. A repeated‐measures analysis of covariance (ANCOVA) was used to examine the effect of age (between group) and sensory condition (within group), on equilibrium score, changes in O_2_Hb and HHb and laterality index, controlling for education as a covariate. For changes in O_2_Hb and HHb, separate ANCOVAs were conducted for left and right hemispheres. Greenhouse–Geisser correction was applied if the assumption of sphericity was violated. Where significant interactions were found, Bonferroni adjustment was applied to post hoc pairwise comparisons. Linear regressions were used to determine whether the change in overall change in O_2_Hb of the PFC was associated with balance, as indexed by the equilibrium score at each sensory condition for both age‐groups. Regression models were additionally adjusted for education level and MMSE score. Fisher's *r*‐to‐*z* transformation compared whether the association between O_2_Hb and equilibrium score differed between young and older adults. Cohen's d effect sizes were compared between the means of the subsample and main study results for both O_2_Hb and HHb (see Supporting Information Figure S2 and Table S1 for results). Alpha level of *p* < 0.05 was used to determine the level of significance. Analyses were conducted using SPSS Statistics V.22.0 (IBM Corp, NY, USA).

## 3 RESULTS

### 3.1 Participant characteristics

Table 1 displays differences in demographics between the young and older adults. The participants in the young group had a higher level of education than those in the older group. No differences in weight, height, or MMSE scores were observed.

**Table 1:** Demographic data for young and older participants

	Young (*n* = 20)	Older (*n* = 18)	*T* test *p*‐values
Gender (M/F)	12/8	10/8	**–**
Age (years)	21.5 ± 3.5	69.5 ± 3.4	**<0.001**
Height (cm)	176.3 ± 12.6	170.6 ± 11.5	0.75
Weight (kg)	74.7 ± 17.4	77.4 ± 15.7	0.87
MMSE score (/30)	29.5 ± 0.9	27.7 ± 1.0	0.66
Education status (years)	15.7 ± 2.3	10.4 ± 3.1	**0.04**

Boldface indicates between‐group significances of *p*<0.05.

### 3.2 Balance performance (equilibrium score)

All equilibrium scores for young and older adults are shown in Table 2. There was an effect for CONDITION (*F*_2.60,91.14_ = 1062.48, *p* < 0.001) and CONDITION x AGE interaction (*F*_3.88,135.68_ = 129.67, *p* < 0.001). Compared to condition 1 (eyes open, unmoving surface, and visual surround) both younger and older adults showed decreases in equilibrium scores for conditions 2 through to 6 (eyes closed conditions and those using the sway‐referenced surface; all *p* < 0.001). For conditions 3–6 (sway‐referenced surface), older adults showed a greater decrease (*p* < 0.001) in equilibrium scores when compared to the younger group.

**Table 2:** Means ± *SD* for equilibrium scores for both young and older adults

Condition	Equilibrium scores		Between‐group *p*‐values
	Young	Old	
1	96.00 ± 1.62	94.56 ± 2.23	**0.027**
2	94.30 ± 1.34	93.89 ± 2.00	0.457
3	94.05 ± 1.32	89.56 ± 2.36	**<0.001**
4	84.60 ± 1.70	80.89 ± 1.60	**<0.001**
5	69.90 ± 2.75	62.17 ± 1.65	**<0.001**
6	65.70 ± 2.18	58.44 ± 2.55	**<0.001**

### 3.3 Changes in _L_DLPFC and _R_DLPFC between sensory conditions

**Figure 2:**
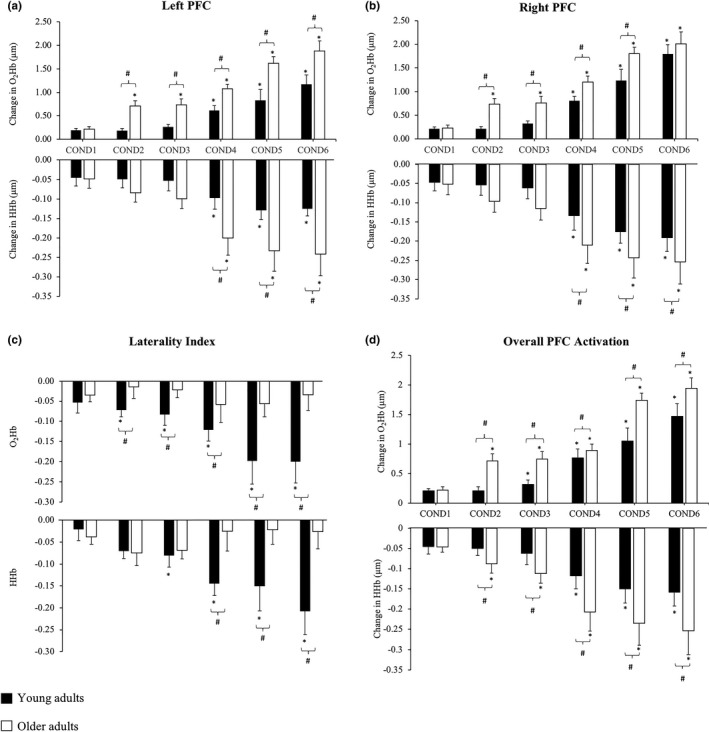
Changes in (a) _L_DLPFC **,** (b) _R_DLPFC, (c) laterality index, and (d) overall DLPFC activation during the six sensory conditions for younger (black columns) and older (white columns) adults. (*) indicates significance *p* < 0.05 from COND1, while (#) indicates significance of *p *< 0.05 between young and older adults

Figure 2a,b shows the changes in O_2_Hb and HHb in the _L_DLPFC and _R_DLPFC respectively during each of the six SOT sensory conditions for younger and older adults. For the _L_DLPFC, a main effect for CONDITION and an AGE x CONDITION interaction was observed in both O_2_Hb (CONDITION = *F*_3.58,125.23_ = 60.33, *p* < 0.001; AGE x CONDITION = *F*_3.58,125.23_ = 25.35, *p* < 0.001) and HHb (CONDITION = *F*_3.58,125.23_ = 15.55, *p* < 0.001; AGE x CONDITION = *F*_3.58,125.23_ = 12.45, *p* < 0.001). No significant difference was observed for condition 1 between younger and older adults (*p* = 0.154); however, from conditions 2 to 6, older adults had a significantly greater increase (*p* < 0.001) in O_2_Hb compared to younger adults and compared to condition 1. Additionally, a significantly greater increase (*p* < 0.001) in O_2_Hb was observed in younger adults from conditions 4 to 6 compared to condition 1. For HHb, no significant differences were observed between conditions 1 to 3 and between age‐groups. However, from conditions 4 to 6, both younger and older adults had a significant reduction in HHb compared to condition 1 (all *p* < 0.001) and between each other (*p* < 0.001).

An effect for CONDITION and AGE x CONDITION interaction was observed in the _R_DLPFC for O_2_Hb (CONDITION = *F*_3.58,125.23_ = 66.64, *p* < 0.001; AGE x CONDITION = *F*_3.58,125.23_ = 10.54, *p* < 0.001) and HHb (CONDITION = *F*_3.58,125.23_ = 14.54, *p* < 0.001; AGE x CONDITION = *F*_3.58,125.23_ = 11.58, *p* < 0.001). In the _R_DLPFC, no significant difference was found in condition 1 (*p* = 0.177) and condition 6 (*p* = 0.105) between younger and older adults. However, from conditions 2 to 5, a significantly greater increase (*p* < 0.001) in O_2_Hb was observed in older compared to younger adults. Compared to condition 1, a greater increase (*p* < 0.001) in O_2_Hb was observed from conditions 2 to 6, while younger adults showed a significant increase (*p* < 0.001) in O_2_Hb on from conditions 4 to 6. For HHb, significant between‐group differences were observed only from conditions 4 to 6, with older adults demonstrating a greater reduction (*p* < 0.01) in HHb compared to younger adults. Compared to condition 1, a significant reduction (*p* < 0.01) in HHb was observed in younger and older adults from conditions 4 to 6, while conditions 2 and 3 were not significantly different from condition 1 in both groups.

### 3.4 Changes in laterality index between conditions

Figure 2c shows the laterality index for O_2_Hb and HHb between sensory conditions. An effect of CONDITION and AGE x CONDITION interaction was found in O_2_Hb (CONDITION = *F*_3.58,125.23_ = 35.67, *p* < 0.001; AGE x CONDITION = *F*_3.58,125.23_ = 21.33, *p* < 0.001) and HHb (CONDITION = *F*_3.58,125.23_ = 21.45, *p* < 0.001; AGE x CONDITION = *F*_3.58,125.23_ = 12.54, *p* < 0.001). In older adults, the laterality index for O_2_Hb and HHb showed no differences across all conditions. However, in younger adults, the laterality index for O_2_Hb in conditions 2–6 were significantly lower (i.e., toward −1, indicating _R_DLPFC lateralization) compared to condition 1 (*p* < 0.001), and compared to older adults (all *p* < 0.001). For HHb, within‐group comparisons showed that the laterality index in younger adults was lower (indicating a greater _R_DLPFC lateralization) from conditions 3 to 6 compared to condition 1 (*p* < 0.001), while between‐group comparisons showed a significant _R_DLPFC lateralization effect from conditions 4 to 6 when compared to older adults (*p* < 0.001).

### 3.5 Changes in overall DLPFC activation between conditions

Figure 2d shows the overall change in O_2_Hb and HHb of the DLPFC between sensory conditions. For overall O_2_Hb, there was an effect for condition (*F*_3.58,125.23_ = 62.36, *p* < 0.001) and condition by age interaction (*F*_3.58,125.23_ = 20.93, *p* < 0.001). During condition 1, no differences between O_2_Hb levels in younger and older adult were observed (*p* = 0.160); however, older adults had greater changes in O_2_Hb levels during conditions 2 through to 6 (*p* < 0.001) compared to young adults. Compared to sensory condition 1, older adults had an increase in O_2_Hb for all conditions (*p* < 0.001), whereas young adults had increases in conditions 3– 6 (all *p* < 0.001).

For overall HHb, there was a condition (*F*_3.88,135.68_ = 4.76, *p* = 0.001) and condition by age interaction (*F*_3.88,135.68_ = 5.89, *p* < 0.001). There were no differences in change in HHb between older adults in condition 1 (*p* = 0.665); however, older adults had a greater decrease in HHb in conditions 2 through to 6. Compared with condition 1, no changes in HHb for condition 2 and 3 were observed for young adults (*p* = 0.999). Younger adults had a decrease in HHb levels in conditions 4, 5, and 6 (*p* < 0.001), whereas older adults had decreases in HHb for all conditions (*p* < 0.001). Overall, no difference in overall O_2_Hb and HHb was observed between the findings from the subsample study and main study (see Supporting Information Appendix S1).

### 3.6 The relationship between changes in O_2_Hb, HHb and balance performance

**Figure 3:**
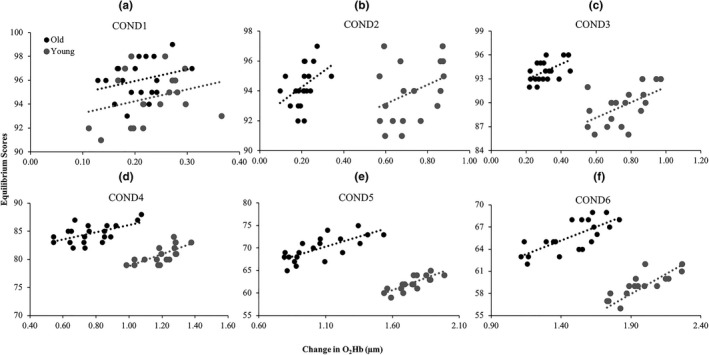
The association between overall O_2_Hb of both the left and right DLPFC and equilibrium scores in young and older adults across all conditions

Figure 3 shows the relationship between overall change in *O*_*2*_ *Hb* and balance performance. For sensory conditions 1 and 2, there was no association between the change in overall change in O_2_Hb levels and equilibrium scores (Table 3). For sensory conditions 3 through to 6, a greater increase in O_2_Hb levels was associated with higher equilibrium scores for both younger and adults, with no differences in this relationship between groups. Additional adjustment for education status and MMSE score did not materially affect these results. Further, no associations between HHb and equilibrium scores (Table 4) were observed in all six sensory conditions.

**Table 3:** Association between the change in overall O_2_Hb and equilibrium score for young and older adults at each sensory condition

Condition	β (95%CI)	*R* ^2^	*p*	β (95%CI)	*R* ^2^	*p*	Fisher's *r* to *z*	*p*
	Young			Old				
1	10.16 (−7.49 to 27.80)	0.08	0.24	9.55 (−7.68 to 26.77)	0.08	0.26	−0.02	0.98
2	10.40 (−1.02 to 21.81)	0.17	0.07	6.31 (−2.05 to 14.67)	0.14	0.13	0.13	0.90
3	9.55 (2.22 to 16.89)	0.29	**0.01**	9.61 (1.73 to 17.49)	0.29	**0.02**	0.91	0.36
4	6.37 (1.66 to 11.09)	0.31	**0.01**	10.48 (4.76 to 16.19)	0.49	**0.001**	−0.66	0.51
5	8.85 (4.72 to 12.99)	0.53	**<0.001**	10.79 (6.41 to 15.16)	0.63	**<0.001**	−0.45	0.65
6	7.30 (3.91 to 10.69)	0.53	**<0.001**	10.84 (5.77 to 15.92)	0.56	**<0.001**	−0.13	0.90

Crude model.

Results adjusted for education and MMSE were not materially different.

Values in boldface denote significance *p *< 0.05.

**Table 4:** Association between the change in overall HHb and equilibrium score for young and older adults at each condition

Condition	β (95%CI)	*R* ^2^	*p*	β (95%CI)	*R* ^2^	*p*
	Young			Old		
1	−2.45 (−34.05 to 29.16)	0.002	0.873	−9.41 (−52.67 to 33.83)	0.013	0.651
2	−2.22 (−26.3 to 21.87)	0.002	0.849	19.08 (−19.86 to 58.02)	0.063	0.314
3	8.85 (−14.05 to 31.76)	0.035	0.427	23.21 (−18.04 to 64.47)	0.082	0.250
4	−5.21 (−28.26 to 17.85)	0.012	0.641	−2.41 (−21.49 to 16.66)	0.005	0.792
5	−19.03 (−68.18 to 30.11)	0.036	0.426	−3.64 (−20.34 to 13.05)	0.013	0.650
6	6.28 (−26.69 to 40.34)	0.010	0.674	18.34 (−2.16 to 38.38)	0.184	0.076

Crude model.

Results adjusted for education and MMSE were not materially different.

## 4 DISCUSSION

This study compared the age‐related changes in hemodynamic response of the bilateral DLPFC during the SOT. Our main findings showed that in both age‐groups, an increase in bilateral DLPFC activation was associated with better balance performance as the level of sensory complexity increased. This was characterized by an increase in bilateral DLPFC O_2_Hb in both age‐groups for all eyes closed conditions and those using the sway‐referenced surface, which was greater in older compared to younger adults. Furthermore, under sensory conditions with increasing complexity, older adults exhibited poorer balance compared to the younger age‐group. Additionally when comparing the left and right DLPFC in both groups, older adults showed greater bilateral activation of the DLPFC during more complex sensory tasks compared to younger adults. This may indicate a greater allocation of cognitive resources in older adults that is compensatory in nature, to maintain balance performance.

In line with previous studies, age‐related increases in postural sway have been reported under conditions that disrupt visual or somatosensory cues (Faraldo‐Garcia, Santos‐Perez, Crujeiras, & Soto‐Varela, 2016; Liaw, Chen, Pei, Leong, & Lau, 2009). In particular, a study by Camicioli et al. (1997) showed that older adults were more impaired with the presence of inaccurate proprioceptive information (while maintaining accurate visual cues), suggesting an increased dependence on proprioceptive cues over visual cues with age. Their results were supported by a recent study by Wiesmeier, Dalin, and Maurer (2015), which suggested that the greater reliance of proprioceptive cues with age may represent some form of compensation strategy in older adults. In our study, we showed in condition 1 (eyes open) that older adults had lower scores compared to young adults; however, in condition 2 (eyes closed), no difference in balance performance were observed. This paradoxical finding was indeed surprising as we initially hypothesized a reduction in balance performance with increasing sensory complexity. However, we further observed that the increase in bilateral DLPFC O_2_Hb was significantly greater during condition 2 compared to condition 1 in older adults, and this increase in DLPFC activity, perhaps as a compensation strategy, may in part explain the attenuation in balance performance declines in condition 2 for older adults. However, in all other conditions (3–6) where either inaccurate proprioceptive and/or vestibular cues were presented, balance performance was significantly impaired in older adults. Indeed, a recent systematic review by de Dieuleveult et al. (2017) indicated that older adults relied more on multisensory information in order to accurately perform activities of daily living. However, in tasks that require increased cognitive loading (i.e., dual‐tasking or distractions), or when inaccurate information was presented, task performance in older adults was greatly impacted. Taken in the context of a balance task, poorer balance performance with increasing complexity of sensory disruption is likely to be indicative of an age‐related deficit in central integration of multiple sensory inputs (Camicioli et al., 1997).

The main finding from this study demonstrated that compared to younger adults, older adults had greater bilateral DLPFC activation (increase in O_2_Hb and decrease in HHb) particularly during more complex balance tasks (see Figure 2a,b,d), while younger adults showed greater lateralization to the _R_DLPFC with increased sensory demands. These findings are in general agreement with previous fNIRS studies (Karim et al., 2013; Lin et al., 2017; Mihara et al., 2008) demonstrating that the PFC and temporal‐parietal regions were engaged during active balancing, which was thought to be involved with allocation of attentional demands in standing postural control. While our data showed that level of bilateral DLPFC activation increased with sensory complexity, it was also observed that the magnitude of bilateral DLPFC activation was greater for the eyes closed conditions and those using the sway‐referenced surface in older adults. In other neuroimaging studies, increased cortical activity with aging was commonly reported. For example, a study by Zwergal et al. (2012) using fMRI showed an age‐related increase in cortical activity of the vestibular and visual cortices during imaginary gait. Similarly, Lin et al. (2017) recently demonstrated an age‐related increase in the frontal–lateral and occipital regions during an abbreviated version of the SOT in middle‐aged and older adults; however, they reported no differences in balance performance between age‐groups. In our study, we observed a greater increase in bilateral DLPFC activation for older adults, which was concomitant with the complexity of the balance task in conditions 3–6. In condition 2, the equilibrium score in both young and older adults was similar; however, the increase in bilateral DLPFC activation was significantly greater in older adults. From a motor control perspective, the DLPFC has been implicated in planning of action motor sequences and allocating attentional resources (Kaller, Rahm, Spreer, Weiller, & Unterrainer, 2011; Unterrainer & Owen, 2006). Therefore, the greater increase in bilateral DLPFC activation with greater sensory demands during the balance tasks in older compared to younger adults may be indicative of greater reliance on executive function, indirect locomotor pathways (Hamacher, Herold, Wiegel, Hamacher, & Schega, 2015; Herold, Wiegel et al., 2017; Wittenberg et al., 2017), and reduced automaticity in balance control (Paul, Ada, & Canning, 2013). The indirect locomotor pathway forms part of the corticobasal ganglia network that is involved with preventing unwanted muscle contractions from competing with voluntary movements (Nambu, 2004).

While we observed a greater increase in bilateral DLPFC activation in older adults that was associated with increased sensory demands, the greater right lateralized effect of DLPFC activation in younger adults suggests a task‐specific reliance of the _R_DLPFC during postural control. In young adults, the inhibition of unwanted movements may have involved a greater involvement of right‐hemisphere brain regions such as the right inferior and superior frontal gyri that are functionally responsible for response inhibition, attention control, and spatial self‐awareness (Goldberg, Herel, & Malach, 2006; Hampshire, Chamberlain, Monti, Duncan, & Owen, 2010). Conversely in older adults, we showed a lower right lateralized effect and by an overall greater increase in bilateral activation of the DLPFC. This reduction in hemispheric asymmetry has previously been proposed by Cabeza (2002) in the hemispheric asymmetry reduction in older adult (HAROLD) model, which suggests that the shift from a lateralized to more symmetrical pattern of DLPFC activation may be compensatory in nature, where greater allocation of resources is required to perform a cognitive task as we age (Grady, McIntosh, & Craik, 2005; Rajah, Languay, & Valiquette, 2010). Additionally, there are further suggestions that the right hemisphere, as depicted by the right hemi‐aging model (Dolcos, Rice, & Cabeza, 2002), may be more susceptible to age‐related declines in cognitive processing, which may help to explain this shift toward an increase in bilateral DLPFC activation in older adults. Taken together, our results in older adults suggest a pattern of overactivity of the bilateral DLPFC that may be a result of greater attentional and resource allocation requirements, and inefficiencies in _R_DLPFC processing of sensory information.

While the magnitude of bilateral DLPFC activation was associated with balance performance in both young and older adults, several limitations have to be acknowledged. We were unable to measure hemodynamic changes in other brain regions such as the occipital, parietal, and temporal areas that have previously been suggested to be implicated in postural control (Karim et al., 2013; Lin et al., 2017). Certainly, increased activation in sensory brain regions during active balance could potentially result in increases in PFC activation to consolidate information from sensory regions. Compared to previous fNIRS studies (Karim et al., 2013; Lin et al., 2017), our study also used a lower number of fNIRS channels. However, we chose to specifically focus all channels solely on the DLPFC and were able to detect significant age‐related changes in hemodynamic responses during the SOT. A further limitation of this study was the omission of short‐separation channels to account for extracerebral blood flow or to exclude any systemic changes that may have occurred. However, since O_2_Hb signals are more affected by extracerebral/systemic physiological changes than HHb (Kirilina et al., 2012), and since O_2_Hb changes were corroborated by HHb changes, we are confident that the differences in DLPFC activation derived from both O_2_Hb and HHb changes are a true representation of cortical activation.

Our study provided evidence for a strong link between the functioning of the DLPFC and postural control, particularly during complex sensory conditions. Our results also showed that bilateral DLPFC activation during postural control increased as a function of aging, which is likely to be indicative of increased attentional resources being used to maintain balance. Taken together, increased bilateral DLPFC activation may act to compensate for balance performance decrements that occur due to both aging and task difficulty. It remains to be seen whether targeted interventions such as exercise or neurocognitive training may lead to improved DLPFC functioning and whether such changes may elicit performance gains in balance performance in older adults.

## CONFLICT OF INTERESTS

The authors declare no conflict of interests.
