# Supplementary material for: Sensory manipulation results in increased dorsolateral prefrontal cortex activation during static postural balance in sedentary older adults: An fNIRS study
Source: Brain Behav. 2018 Sep 19;8(10):e01109. doi: 10.1002/brb3.1109 (PMC6192391; doi:10.1002/brb3.1109)
Supplement: Supplementary file 1 [file BRB3-8-e01109-s001.docx]

**Supplementary Data**


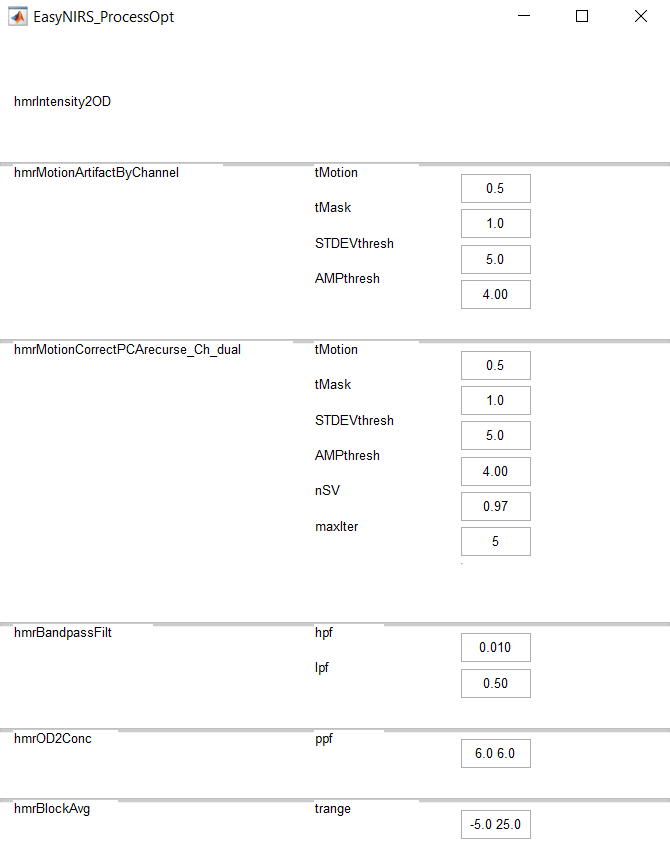


**Figure. 1** HOMER2 data processing pipeline for artefact identification, removal and associated filtering functions.


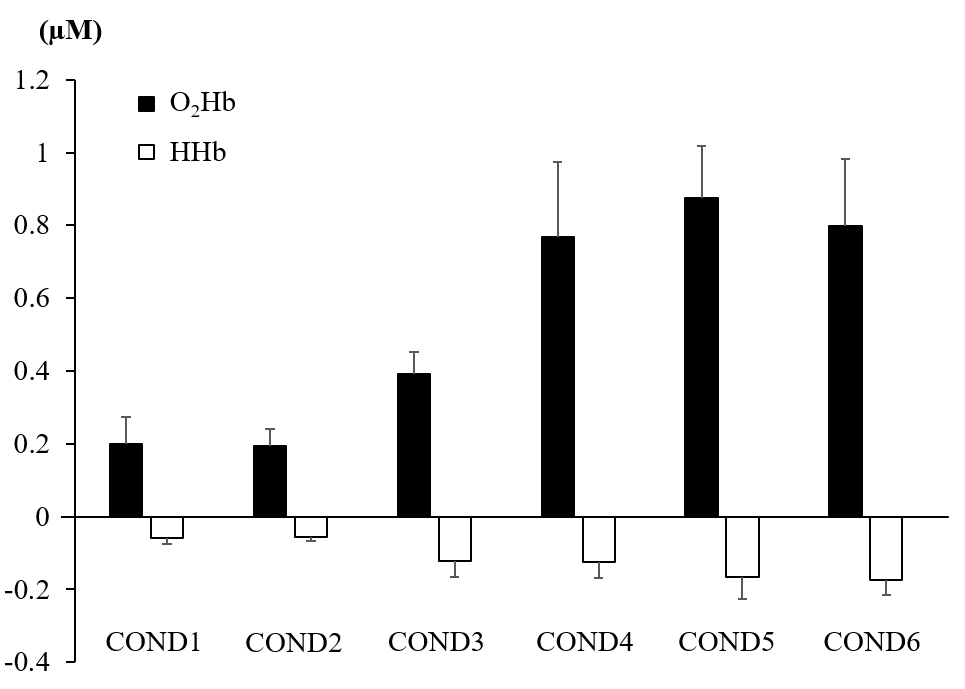


**Figure 2.** Changes in O_2_Hb and HHb from a sub-sample of 5 young healthy adults that performed the 6 sensory conditions in random order.

**Table 1** A Cohen’s *d* effect size comparison between O_2_Hb and HHb results from the sub-sample (n=5) and main study (n=20)

| **Condition** | **Cohens d** | **Lower 95%CI** | **Upper 95%CI** |
| --- | --- | --- | --- |
| *O_2_Hb* |  |  |  |
| COND1 | -0.138 | -1.117 | 0.844 |
| COND2 | -0.192 | -1.171 | 0.791 |
| COND3 | 1.019 | -0.013 | 2.032 |
| COND4 | -0.003 | -0.983 | 0.976 |
| COND5 | -0.834 | -1.834 | 0.183 |
| COND6 | -3.172 | -4.492 | -1.815 |
|  |  |  |  |
| *HHb* |  |  |  |
| COND1 | -0.445 | -1.428 | 0.548 |
| COND2 | -0.156 | -1.135 | 0.826 |
| COND3 | -1.953 | -3.067 | -0.808 |
| COND4 | -0.025 | -1.005 | 0.954 |
| COND5 | -0.333 | -1.315 | 0.654 |
| COND6 | -0.415 | -1.398 | 0.575 |
